# Nivolumab plus chemotherapy in patients with HER2-negative, previously untreated, unresectable, advanced, or recurrent gastric/gastroesophageal junction cancer: 3-year follow-up of the ATTRACTION-4 randomized, double-blind, placebo-controlled, phase 3 trial

**DOI:** 10.1007/s10120-024-01535-0

**Published:** 2024-08-20

**Authors:** Narikazu Boku, Takeshi Omori, Kohei Shitara, Shinichi Sakuramoto, Kensei Yamaguchi, Ken Kato, Shigenori Kadowaki, Kunihiro Tsuji, Min-Hee Ryu, Do-Youn Oh, Sang Cheul Oh, Sun Young Rha, Keun-Wook Lee, Ik-Joo Chung, Sun Jin Sym, Li-Tzong Chen, Jen-Shi Chen, Li-Yuan Bai, Takashi Nakada, Shunsuke Hagihara, Reina Makino, Eiji Nishiyama, Yoon-Koo Kang

**Affiliations:** 1 Department of Oncology and General Medicine, IMSUT Hospital, Institute of Medical Science, University of Tokyo, Tokyo, Japan; 2 Department of Gastroenterological Surgery, Osaka International Cancer Institute, Osaka, Japan; 3 Department of Gastroenterology and Gastrointestinal Oncology, National Cancer Center Hospital East, Kashiwa, Japan; 4 Saitama Medical University International Medical Center, Hidaka, Japan; 5 Department of Gastroenterological Chemotherapy, The Cancer Institute Hospital of Japanese Foundation for Cancer Research, Tokyo, Japan; 6 Division of Gastrointestinal Medical Oncology, National Cancer Center Hospital, Tokyo, Japan; 7 Department of Clinical Oncology, Aichi Cancer Center Hospital, Nagoya, Japan; 8 Department of Medical Oncology, Ishikawa Prefectural Central Hospital, Kanazawa, Japan; 9 Department of Oncology, Asan Medical Center, University of Ulsan College of Medicine, Seoul, South Korea; 10 Department of Internal Medicine, Seoul National University Hospital, Cancer Research Institute, Seoul National University College of Medicine, Seoul, South Korea; 11 Division of Hematology and Oncology, Department of Internal Medicine, College of Medicine, Korea University, Seoul, South Korea; 12 Division of Medical Oncology, Yonsei Cancer Center, Yonsei University Health System, Songdang Institute for Cancer Research, Yonsei University College of Medicine, Seoul, South Korea; 13 Division of Hematology and Medical Oncology, Department of Internal Medicine, Seoul National University Bundang Hospital, Seoul National University College of Medicine, Seongnam, South Korea; 14 Department of Hematology-Oncology, Chonnam National University Hwasun Hospital, Chonnam National University College of Medicine, Hwasun, South Korea; 15 Division of Medical Oncology, Department of Internal Medicine, Gachon University Gil Medical Center, Incheon, South Korea; 16 National Institute of Cancer Research, National Health Research Institutes, and National Cheng Kung University Hospital, National Cheng Kung University, Tainan, Taiwan; 17 Kaohsiung Medical University Hospital, and Center for Cancer Research, Kaohsiung Medical University, Kaohsiung, Taiwan; 18 Division of Hematology and Oncology, Department of Internal Medicine, Linkou Chang Gung Memorial Hospital, Chang Gung University, Taoyuan, Taiwan; 19 Division of Hematology and Oncology, Department of Internal Medicine, China Medical University Hospital, and China Medical University, Taichung, Taiwan; 20 Department of Oncology Clinical Development Planning, Ono Pharmaceutical Co., Ltd., Osaka, Japan; 21 Department of Statistical Analysis, Ono Pharmaceutical Co., Ltd., Osaka, Japan; 22 Department of Medical Affairs, Ono Pharmaceutical Co., Ltd., Osaka, Japan; 23 Department of Oncology, Asan Medical Center, University of Ulsan College of Medicine, 88, Olympic-ro, 43-gil, Songpa-gu, Seoul, 05505 South Korea

**Keywords:** Gastric cancer, Chemotherapy, Nivolumab, Oxaliplatin, Randomized controlled trial

## Abstract

**Background:**

Nivolumab + chemotherapy is now a standard of care for HER2-negative, previously untreated, unresectable or recurrent gastric/gastroesophageal junction cancer (advanced gastric cancer), but long-term follow-up data of clinical trials are limited.

**Methods:**

ATTRACTON-4 was a phase 3, double-blind, placebo-controlled trial in Japan, South Korea, and Taiwan. Patients were randomized to either nivolumab or placebo, both combined with the physician’s choice of SOX (oral S-1 [tegafur–gimeracil–oteracil potassium] + oxaliplatin) or CAPOX (capecitabine + oxaliplatin). We report the primary endpoints—centrally assessed progression-free survival (PFS) and overall survival (OS)—and landmark analyses of OS among patients alive using 3-year follow-up data.

**Results:**

At the cutoff date (May 10, 2021), 17/359 patients in the nivolumab + chemotherapy group and 6/358 in the placebo + chemotherapy group were continuing study treatment. PFS (centrally assessed) was longer in the nivolumab + chemotherapy group (median 10.94 vs. 8.48 months; hazard ratio [HR] 0.67, 95% confidence interval [CI] 0.55–0.82). Although OS did not differ between the two groups (median 17.45 vs. 17.15 months; HR 0.89, 95% CI 0.75–1.05), the landmark analysis of OS, calculating HRs at each landmark time point (every month), was getting numerically better in the nivolumab + chemotherapy group over time. Approximately 80% of patients who achieved complete response in the nivolumab + chemotherapy group were alive at 3 years. No new safety signals or major late-onset select treatment-related adverse events were observed for nivolumab + chemotherapy.

**Conclusion:**

This 3-year follow-up of ATTRACTION-4 confirmed the long-term clinical benefit and manageable safety of nivolumab + chemotherapy in patients with previously untreated advanced gastric cancer.

**Trial registration:**

NCT02746796

**Supplementary Information:**

The online version contains supplementary material available at 10.1007/s10120-024-01535-0.

## Introduction

It was estimated that there were just over 1 million new cases of gastric cancer worldwide in 2020, with an age-adjusted incidence of 11.1 per 100,000 people [1]. It has also been reported that the cumulative risk of gastric cancer is higher in East Asia than in other regions [2]. However, Asian patients with unresectable or recurrent (advanced) gastric cancer tended to show better survival after treatment than non-Asian patients in clinical trials [3–9].

Fluoropyrimidine- and platinum-based chemotherapy was the first-line standard of care for HER2-negative, advanced gastric cancer and this treatment has not changed substantially over the past decade. However, nivolumab plus chemotherapy demonstrated a significant clinical benefit in the first-line setting in two phase 3 trials: CheckMate 649 [10] and ATTRACTION-4 [11]. On the one hand, ATTRACTION-4, which was performed in Japan, South Korea, and Taiwan, showed that nivolumab plus SOX (oral S-1 [tegafur–gimeracil–oteracil potassium] plus oxaliplatin) or CAPOX (capecitabine plus oxaliplatin) chemotherapy significantly improved progression-free survival (PFS), but not overall survival (OS), as first-line therapy in Asian patients with advanced gastric cancer [11]. On the other hand, CheckMate 649, which was performed in Asia, Australia, Europe, North America, and South America, showed that nivolumab plus XELOX (capecitabine plus oxaliplatin; CAPOX) or FOLFOX (fluorouracil, leucovorin, and oxaliplatin) chemotherapy improved OS and PFS vs. chemotherapy alone in the overall cohort, and in patients with a programmed cell death ligand 1 (PD-L1) combined positive score (CPS) of ≥ 1 or ≥ 5 (minimum follow-up 12.1 months) [10].

Nivolumab plus chemotherapy has now been approved in > 50 countries as first-line therapy for patients with advanced gastric cancer, including the United States (April 2021) [12], South Korea (June 2021), Taiwan (October 2021), the European Union (October 2021), and Japan (November 2021) [13]. This regimen has since been added as a treatment option into clinical guidelines, including the NCCN Guidelines for Gastric Cancer [14], the Japanese Gastric Cancer Treatment Guidelines 2021 [15], and the Korean Gastric Cancer Guidelines 2023 [16].

In CheckMate 649, nivolumab plus chemotherapy continued to demonstrate a clinically meaningful improvement in efficacy versus chemotherapy alone with an acceptable safety profile after 2 years of follow-up [17]. The 3-year follow-up results of CheckMate 649 also showed a consistent survival benefit of nivolumab [18]. However, the long-term outcomes of Asian patients treated with nivolumab plus chemotherapy remain unknown. Therefore, we performed a 3-year follow-up of ATTRACTION-4, and we report the updated efficacy and safety data at a cutoff date of May 10, 2021. Data from this analysis will provide evidence regarding the long-term durability and safety of nivolumab combined with chemotherapy. Owing to the availability of 3-year follow-up, we also performed landmark analyses of OS over time by assessing the hazard ratio at each landmark time.

## Methods

### Ethics

As previously described [11], this trial was approved by the institutional review board/ethics committee at each participating site and adhered to the Declaration of Helsinki and Good Clinical Practice.

### Patients

Briefly, patients aged ≥ 20 years with histologically confirmed gastric or gastroesophageal junction cancer (unresectable or recurrent) could be enrolled if they had no history of prior treatment except for neoadjuvant/adjuvant chemotherapy completed ≥ 180 days before recurrence. Patients were also required to have at least one measurable or evaluable lesion (per Response Evaluation Criteria in Solid Tumors [RECIST] version 1.1, as detected on computed tomography [CT] or magnetic resonance imaging [MRI] up to 28 days before randomization) and availability of tumor tissue specimens (archival or fresh biopsy) to determine tumor cell programmed death ligand 1 (PD-L1) expression prior to randomization. The other eligibility criteria are described in detail in our previous article [11]. All patients provided written informed consent to participate in the trial.

### Study design

The study was conducted at 130 hospitals in Japan, South Korea, and Taiwan. After selection of the chemotherapy regimen (SOX or CAPOX), patients were randomized (1:1) to treatment with either nivolumab or placebo, in combination with the chosen chemotherapy in 6-week cycles [11]. Randomization was stratified according to PD-L1 expression (tumor proportion score ≥ 1% vs. < 1% or undetermined), Eastern Cooperative Oncology Group performance status (ECOG PS; 0 vs. 1), disease status (unresectable vs. recurrent), and geographical region (Japan vs. South Korea or Taiwan). As a double-blind study, the patients, investigators, and the study sponsors were blinded to the allocated treatment. For patients starting a subsequent therapy, the investigators could request unblinding of the study treatment.

Both treatments were to continue until the investigator judged disease progression (RECIST version 1.1), unacceptable toxicity, or consent withdrawal. It was permitted to reduce the dose of chemotherapy, but not the dose of nivolumab or placebo. If either the study drug (nivolumab/placebo) or chemotherapy was discontinued, the remaining drug could be continued if the patient met the criteria. Furthermore, nivolumab or placebo (but not chemotherapy) could be continued following the first event of disease progression, at the investigator’s judgement and after obtaining written consent from the patient.

Efficacy outcomes comprised PFS, OS, tumor responses (evaluated per RECIST version 1.1), objective response rate (ORR), disease control rate (DCR), duration of response (DOR), and time to response (TTR). Tumor responses and PFS were judged by the investigators and a central review committee. Safety was assessed in terms of treatment-related adverse events (TRAEs) using the National Cancer Institute Common Terminology Criteria for Adverse Events (version 4.0) and MedDRA (version 22.1).

### Statistical analyses

This article reports data obtained at the cutoff date of May 10, 2021. All of the analyses reported here were performed in a post hoc manner, using essentially the same statistical methods as those used in the prior article [11]. The primary endpoints were PFS and OS. PFS was defined as the time from randomization to the date of centrally assessed progressive disease according to RECIST guidelines (version 1.1) or death from any cause, whichever occurred first. OS was defined as the time from randomization to the date of death from any cause.

The efficacy outcomes examined in this 3-year follow-up were PFS, OS, ORR, and DCR. These outcomes were assessed in the intention-to-treat (ITT) population, which included all randomly assigned patients. TTR and DOR were analyzed among patients in the ITT population whose best overall response was a complete response (CR) or partial response (PR). PFS and OS were compared between the two treatment groups using a stratified log-rank test with the stratification factors (PD-L1 expression, ECOG PS, disease status, and geographical region). Hazard ratios (HR) with 95% confidence intervals (CI) were also calculated using the stratified Cox proportional hazards model with the stratification factors. The median times for OS, PFS, and DOR were estimated with the Kaplan–Meier method. The 95% CIs for the median time were calculated using the Brookmeyer and Crowley method with double-logarithmic conversion. The ORR and DCR were compared using the Cochran–Mantel–Haenszel test with the stratification factors.

We also performed a landmark analysis of OS over time by calculating HRs using the unstratified Cox proportional hazards model from the mortality rate after each landmark time point (every month) as the index [19]. Furthermore, the investigator-assessed tumor responses were stratified for 3-year survivors and non-3-year survivors in both groups.

Safety was assessed in all patients who received at least one dose of the assigned study treatment, and was summarized descriptively [11]. For potential immune-related TRAEs, we also determined their timing of onset from the start of study drug administration.

## Results

### Patients

As previously reported, 724 patients were randomized between March 23, 2017, and May 10, 2018, with 362 allocated to nivolumab plus chemotherapy and 362 to placebo plus chemotherapy [11]. Overall, 17 of 359 patients who started nivolumab plus chemotherapy, and 6 of 358 who started placebo plus chemotherapy were continuing study treatment at the data cutoff (Supplementary Fig. 1 in Online Resource 1).

The characteristics of patients (ITT population) are summarized in Table 1, and described in detail previously [11]. Based on the safety analysis set, SOX and CAPOX were used in 229 and 130 patients in the nivolumab plus chemotherapy group, and 230 and 128 patients in the placebo plus chemotherapy group. In these subgroups, the median durations of treatment with nivolumab or placebo, oxaliplatin, S-1, and capecitabine ranged from 4.6 to 7.1 months (Supplementary Table 1 in Online Resource 1).

**Table 1:** Patient characteristics

	Nivolumab plus chemotherapy group (*n* = 362)	Placebo plus chemotherapy group (*n* = 362)
Age, years	64 (25–86)	65 (27–89)
Sex		
Male	253 (70%)	270 (75%)
Female	109 (30%)	92 (25%)
Country		
Japan	198 (55%)	197 (54%)
South Korea	148 (41%)	143 (40%)
Taiwan	16 (4%)	22 (6%)
Eastern Cooperative Oncology Group performance status		
0	195 (54%)	194 (54%)
1	167 (46%)	168 (46%)
Disease status		
Advanced	280 (77%)	279 (77%)
Recurrent	82 (23%)	83 (23%)
Primary sites (anatomical subsites, TNM classification)*		
Gastroesophageal junction cancer	29 (8%)	33 (9%)
Gastric cancer	237 (65%)	238 (66%)
Not available	96 (27%)	91 (25%)
Previous gastrectomy		
No	257 (71%)	258 (71%)
Yes	105 (29%)	104 (29%)
Perioperative chemotherapy		
No	294 (81%)	303 (84%)
Yes	68 (19%)	59 (16%)
Number of organs with metastases		
≤ 1	108 (30%)	105 (29%)
≥ 2	254 (70%)	257 (71%)
Site of metastasis†		
Lymph node	286 (79%)	291 (80%)
Liver	132 (36%)	132 (36%)
Peritoneum	173 (48%)	163 (45%)
Histology		
Intestinal type	139 (38%)	154 (43%)
Diffuse type	192 (53%)	176 (49%)
Other	11 (3%)	12 (3%)
Unknown	20 (6%)	20 (6%)
Tumor cell PD-L1 expression		
< 1%	304 (84%)	306 (85%)
≥ 1%	58 (16%)	56 (15%)
Chemotherapy regimen		
Tegafur–gimeracil–oteracil potassium (S-1) plus oxaliplatin	232 (64%)	232 (64%)
Capecitabine plus oxaliplatin	130 (36%)	130 (36%)

Data are median (minimum to maximum range) or *n* (%)

Reprinted with permission from Kang YK, et al. *Lancet Oncol*. 2022;23(2):234–47. https://doi.org/10.1016/S1470-2045(21)00692-6

*Data for primary sites were collected from patients with advanced gastric cancer; patients with recurrent gastric cancer and patients with advanced gastric cancer whose primary sites were not reported or unknown were summarized as not available

^†^More than one site could be assigned

Disease progression was the most common reason for discontinuing nivolumab or placebo in both groups, accounting for 61.0% of patients in the nivolumab plus chemotherapy group and 72.6% in the placebo plus chemotherapy group (Supplementary Table 1 in Online Resource 1). Subsequent therapies included radiotherapy, surgery, and pharmacotherapies. Nivolumab was more frequently used as subsequent therapy in the placebo plus chemotherapy group (30.7%) than in the nivolumab plus chemotherapy group (11.1%) (Supplementary Table 1 in Online Resource 1).

### Progression-free survival

**Fig. 1:**
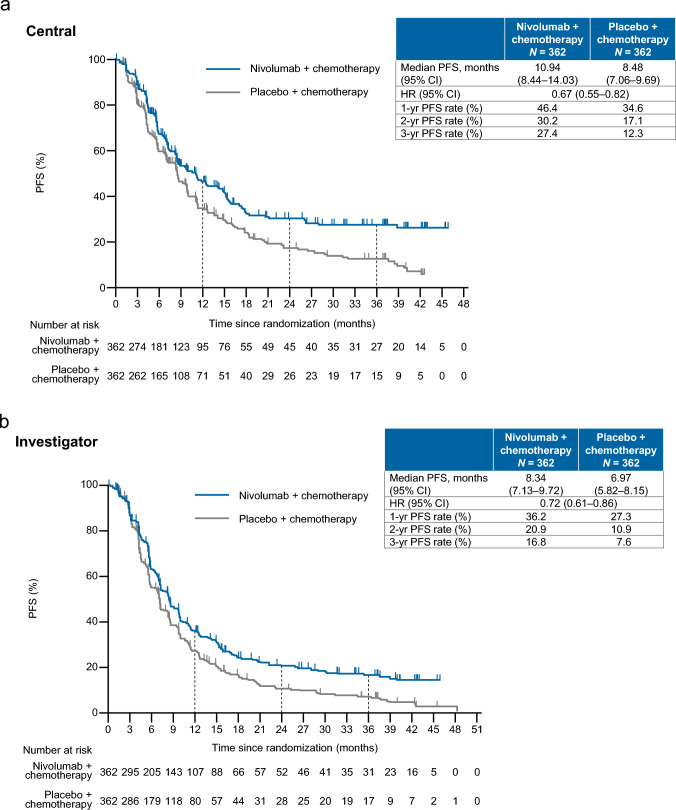

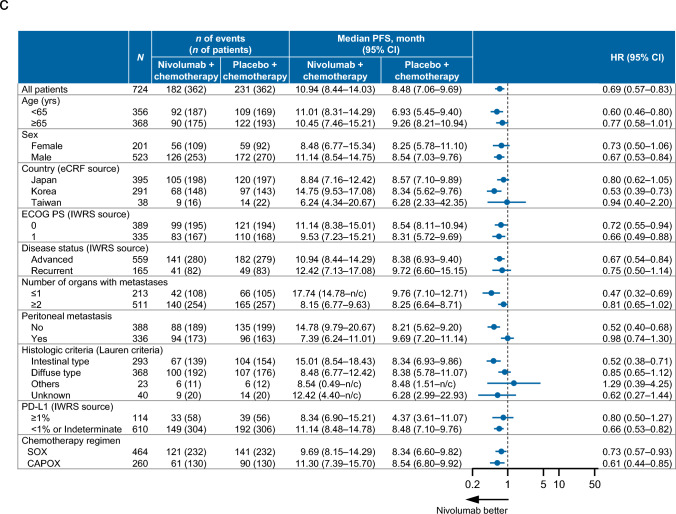
**a** Centrally assessed PFS. **b** Investigator-assessed PFS. **c** Forest plot analysis of centrally assessed PFS. *CAPOX* capecitabine plus oxaliplatin, *CI* confidence interval, *ECOG PS* Eastern Cooperative Oncology Group performance status, *eCRF* electronic case report form, *HR* hazard ratio, *IWRS* interactive web response system, *n/c* not countable, *PD-L1* programmed death ligand 1, *PFS* progression-free survival, *SOX* S-1 (tegafur–gimeracil–oteracil potassium) plus oxaliplatin

At the data cutoff, the median PFS (centrally assessed) was 10.94 months in the nivolumab plus chemotherapy group vs. 8.48 months in the placebo plus chemotherapy group, with a HR of 0.67 (95% CI 0.55–0.82) showing more favorable PFS in the nivolumab plus chemotherapy group, with a 3-year PFS rate of 27.4% (vs. 12.3%) (Fig. 1a). The 1-, 2-, and 3-year PFS rates were consistently greater in the nivolumab plus chemotherapy group. The investigator-assessed PFS also tended to be greater in the nivolumab plus chemotherapy group (median: 8.34 vs. 6.97 months; HR 0.72, 95% CI 0.61–0.86), with a 3-year PFS rate of 16.8% (vs. 7.6%) (Fig. 1b).

In the forest plot analysis, the centrally assessed PFS was more favorable with nivolumab plus chemotherapy (95% CIs did not cross 1) in most subgroups, including patients with tumor cell PD-L1 expression < 1% or indeterminate (Fig. 1c).

### Overall survival

**Fig. 2:**
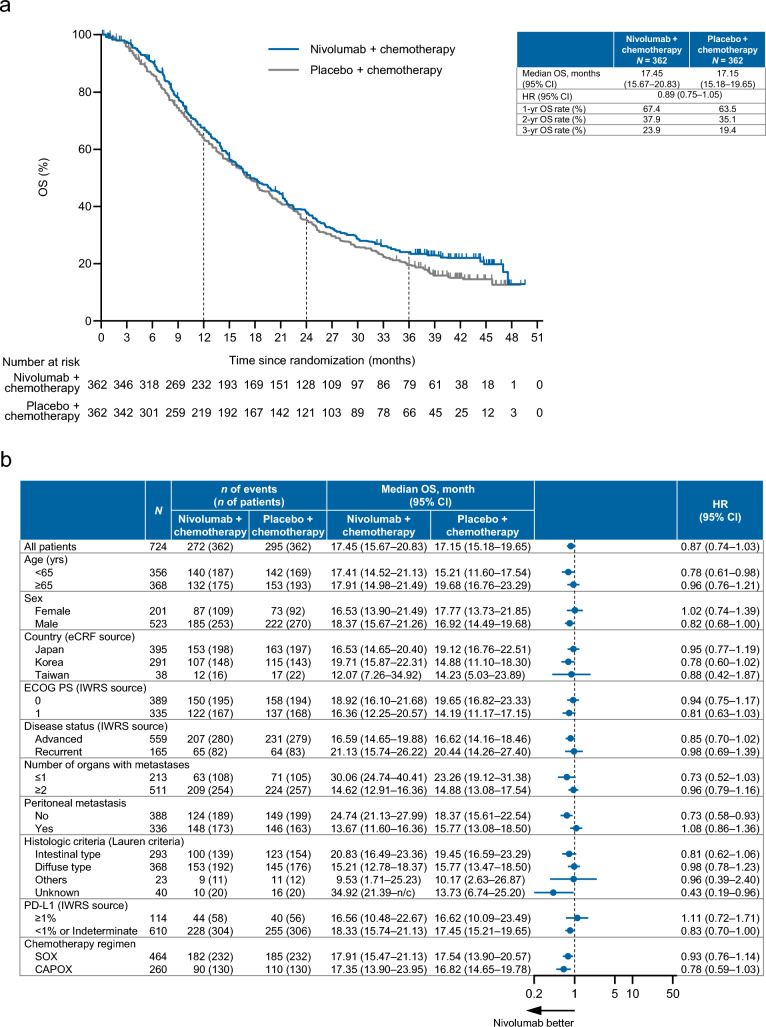

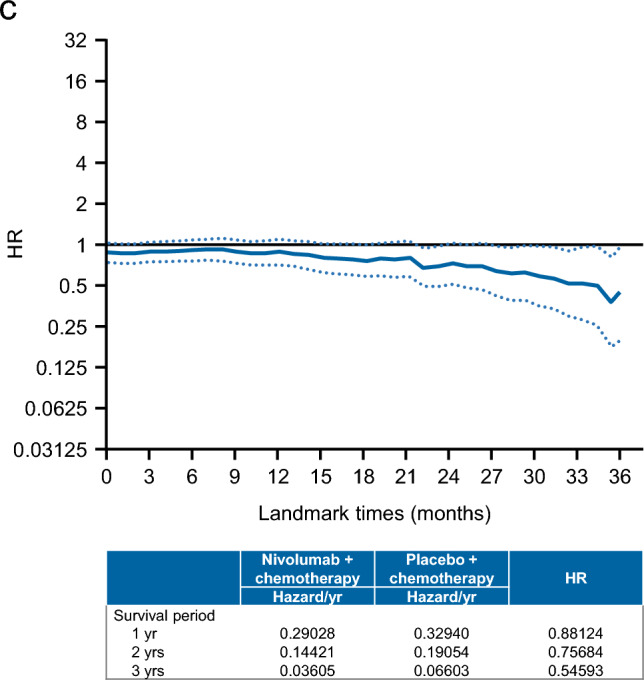
**a** OS. **b** Forest plot analysis of OS. **c** Landmark analysis of OS. *CAPOX* capecitabine plus oxaliplatin, *CI* confidence interval, *ECOG PS* Eastern Cooperative Oncology Group performance status, *eCRF* electronic case report form, *HR* hazard ratio, *IWRS* interactive web response system, *n/c* not countable, *OS* overall survival, *PD-L1* programmed death ligand 1, *SOX* S-1 (tegafur–gimeracil–oteracil potassium) plus oxaliplatin

As shown in Fig. 2a, the OS did not differ between the two groups, with a median OS of 17.45 months in the nivolumab plus chemotherapy group vs. 17.15 months in the placebo plus chemotherapy group (HR 0.89, 95% CI 0.75–1.05). The OS rates were also not markedly different, with 3-year rates of 23.9% and 19.4%, respectively. Among 3-year survivors, an immune checkpoint inhibitor was administered as a subsequent therapy to 11.4% (9/79) of patients in the nivolumab plus chemotherapy group and 48.5% (32/66) in the chemotherapy group.

According to the forest plot analysis, OS tended to be more favorable for nivolumab plus chemotherapy among patients aged < 65 years, patients without peritoneal metastasis, and patients with an unknown histologic type (Fig. 2b). OS did not differ between the two groups regardless of whether chemotherapy comprised SOX or CAPOX.

In the landmark analysis, the hazards/year at the landmark times of 1, 2, and 3 years were 0.29, 0.14, and 0.04, respectively, in the nivolumab plus chemotherapy group. The corresponding hazards/year were 0.33, 0.19, and 0.07 in the placebo plus chemotherapy group. The HRs at the landmark times of 1, 2, and 3 years were 0.88, 0.76, and 0.55, respectively, indicating a tendency toward more improved survival in the long term in the nivolumab plus chemotherapy group (Fig. 2c).

### Tumor responses

The centrally assessed responses were better in the nivolumab plus chemotherapy group, with an ORR of 57.5% vs. 47.8% in the placebo plus chemotherapy group, and a greater proportion of patients with CR in the former group (20.7% vs. 13.8%) (Table 2). Although the ORR was identical to that reported in the previous manuscript, responses in five cases of PR were improved to CR in the nivolumab plus chemotherapy group and two cases of PR were improved to CR in the placebo plus chemotherapy group at the latest cutoff. The centrally assessed DCR was 72.1% in the nivolumab plus chemotherapy group and 68.5% in the placebo plus chemotherapy group. The TTR was 1.4 months in both groups.

**Table 2:** Centrally assessed objective responses

	Nivolumab plus chemotherapy *N* = 362	Placebo plus chemotherapy *N* = 362
ORR (CR + PR),* n* (%) [95% CI]	208 (57.5) [52.2–62.6]	173 (47.8) [42.5–53.1]
Best overall response, *n* (%)		
CR	75 (20.7)	50 (13.8)
PR	133 (36.7)	123 (34.0)
SD	53 (14.6)	75 (20.7)
PD	25 (6.9)	46 (12.7)
NE	76 (21.0)	68 (18.8)
DCR (CR + PR + SD), *n* (%) [95% CI]	261 (72.1) [67.2–76.7]	248 (68.5) [63.4–73.3]
TTR, months, median (range)	1.4 (1.0–6.9)	1.4 (1.0–15.3)

*CI* confidence interval, *CR* complete response, *DCR* disease control rate, *NE* not evaluable, *ORR* objective response rate, *PD* progressive disease, *PR* partial response, *SD* stable disease, *TTR* time to response

**Fig. 3:**
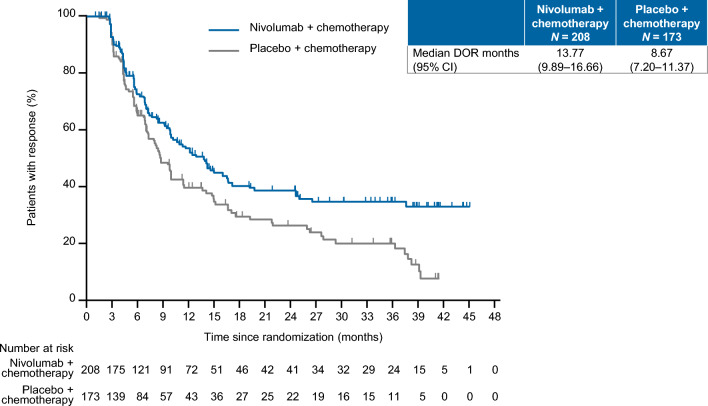
DOR in patients whose best objective response was CR or PR. *CI* confidence interval, *CR* complete response, *DOR* duration of response, *PR* partial response

As shown in Fig. 3, the DOR among patients with CR or PR was longer in the nivolumab plus chemotherapy group with a median of 13.77 months vs. 8.67 months in the placebo plus chemotherapy group.

The investigator-assessed objective responses, which are summarized in Table 3, were numerically similar to the centrally assessed responses. Table 3 also shows the investigator-assessed responses for 3-year survivors and non-survivors. Overall, among patients who achieved CR (investigator’s assessment), 78.6% (11/14) in the nivolumab plus chemotherapy group and 36.4% (4/11) in the placebo plus chemotherapy group were alive at 3 years. Comparable findings were obtained for 3-year survivors based on centrally assessed responses (data not shown).

**Table 3:** Investigator-assessed objective responses (ITT, 3-year survivors, non-3-year survivors)

	ITT		3-year survivors		Non-3-year survivors	
	Nivolumab plus chemotherapy *N* = 362	Placebo plus chemotherapy *N* = 362	Nivolumab plus chemotherapy *N* = 79	Placebo plus chemotherapy *N* = 66	Nivolumab plus chemotherapy *N* = 283	Placebo plus chemotherapy *N* = 296
ORR (CR + PR), *n* (%)	227 (62.6)	195 (53.9)	67 (84.8)	52 (78.8)	160 (56.5)	143 (48.3)
Best overall response, *n* (%)						
CR	14 (3.9)	11 (3.0)	11 (13.9)	4 (6.1)	3 (1.1)	7 (2.4)
PR	213 (58.8)	184 (50.8)	56 (70.9)	48 (72.7)	157 (55.5)	136 (45.9)
SD	75 (20.7)	112 (30.9)	8 (10.1)	11 (16.7)	67 (23.7)	101 (34.1)
PD	37 (10.2)	34 (9.4)	1 (1.3)	2 (3.0)	36 (12.7)	32 (10.8)
NE	23 (6.4)	21 (5.8)	3 (3.8)	1 (1.5)	20 (7.1)	20 (6.8)
DCR (CR + PR + SD), *n* (%)	302 (83.4)	307 (84.8)	75 (94.9)	63 (95.5)	227 (80.2)	244 (82.4)

*CR* complete response, *DCR* disease control rate, *ITT* intention-to-treat, *NE* not evaluable, *ORR* objective response rate, *PD* progressive disease, *PR* partial response, *SD* stable disease

### Safety

Table 4 summarizes the safety data in terms of the TRAEs that occurred during the study, including the serious TRAEs, TRAEs leading to treatment discontinuation, and TRAEs that led to a dose delay or reduction in the dose of at least one drug (nivolumab, placebo, or component of chemotherapy). The incidence and types of TRAEs were similar to those reported in the prior analysis [11]. The percentages of patients with Grade 3 TRAEs, including serious Grade 3 TRAEs and Grade 3 TRAEs leading to dose delay or dose reduction, were numerically greater in the nivolumab plus placebo group. The proportions of patients with Grade 4 or 5 TRAEs were similar in both groups. The five most frequent TRAEs in both groups were peripheral sensory neuropathy, nausea, appetite decreased, diarrhea, and platelet count decreased. Immune-related TRAEs, especially endocrine, gastrointestinal, and skin TRAEs, were most common during the early period of treatment (within 0‒3 months), with low incidences during long-term treatment (Supplementary Fig. 2 in Online Resource 1). No new safety signals or major late-onset immune-related TRAEs were observed in the nivolumab plus chemotherapy group (Supplementary Fig. 2 in Online Resource 1).

**Table 4:** Treatment-related adverse events

Patients, *n* (%)	Nivolumab plus chemotherapy *N* = 359				Placebo plus chemotherapy *N* = 358			
	Grade 1–2	Grade 3	Grade 4	Grade 5	Grade 1–2	Grade 3	Grade 4	Grade 5
Any TRAE	141 (39.3)	192 (53.5)	15 (4.2)	3 (0.8)	173 (48.3)	160 (44.7)	14 (3.9)	2 (0.6)
Any serious TRAE	19 (5.3)	60 (16.7)	8 (2.2)	3 (0.8)	16 (4.5)	28 (7.8)	5 (1.4)	2 (0.6)
Any TRAE leading to discontinuation^a^	10 (2.8)	8 (2.2)	3 (0.8)	3 (0.8)	7 (2.0)	5 (1.4)	3 (0.8)	2 (0.6)
Any TRAE leading to dose delay or dose reduction^b^	137 (38.2)	160 (44.6)	10 (2.8)	0	151 (42.2)	128 (35.8)	12 (3.4)	0
TRAEs^c^								
Peripheral sensory neuropathy	187 (52.1)	14 (3.9)	0	0	181 (50.6)	8 (2.2)	0	0
Nausea	171 (47.6)	10 (2.8)	0	0	169 (47.2)	12 (3.4)	0	0
Decreased appetite	159 (44.3)	29 (8.1)	0	0	173 (48.3)	23 (6.4)	0	0
Diarrhea	109 (30.4)	16 (4.5)	0	0	96 (26.8)	14 (3.9)	0	0
Platelet count decreased	109 (30.4)	29 (8.1)	6 (1.7)	0	124 (34.6)	24 (6.7)	9 (2.5)	0
Neutrophil count decreased	86 (24.0)	71 (19.8)	0	0	77 (21.5)	55 (15.4)	2 (0.6)	0
Fatigue	67 (18.7)	5 (1.4)	0	0	70 (19.6)	2 (0.6)	0	0
Vomiting	67 (18.7)	5 (1.4)	0	0	63 (17.6)	3 (0.8)	0	0
White blood cell count decreased	67 (18.7)	10 (2.8)	0	0	51 (14.2)	7 (2.0)	2 (0.6)	0
Malaise	59 (16.4)	0	0	0	61 (17.0)	1 (0.3)	0	0
Stomatitis	59 (16.4)	6 (1.7)	0	0	42 (11.7)	4 (1.1)	0	0
Dysgeusia	58 (16.2)	0	0	0	52 (14.5)	0	0	0
Aspartate aminotransferase increased	50 (13.9)	4 (1.1)	1 (0.3)	0	42 (11.7)	2 (0.6)	0	0
Palmar-plantar erythrodysesthesia syndrome	49 (13.6)	6 (1.7)	0	0	47 (13.1)	4 (1.1)	0	0
Neuropathy peripheral	48 (13.4)	3 (0.8)	0	0	37 (10.3)	10 (2.8)	0	0
Rash	45 (12.5)	2 (0.6)	0	0	14 (3.9)	0	0	0
Constipation	44 (12.3)	1 (0.3)	0	0	26 (7.3)	0	0	0
Pruritus	42 (11.7)	0	0	0	14 (3.9)	0	0	0
Anemia	40 (11.1)	29 (8.1)	0	0	48 (13.4)	19 (5.3)	0	0
Alanine aminotransferase increased	39 (10.9)	2 (0.6)	1 (0.3)	0	30 (8.4)	6 (1.7)	0	0
Blood bilirubin increased	30 (8.4)	5 (1.4)	1 (0.3)	0	21 (5.9)	2 (0.6)	0	0
Infusion-related reaction	27 (7.5)	5 (1.4)	0	0	14 (3.9)	2 (0.6)	0	0
Asthenia	22 (6.1)	6 (1.7)	0	0	21 (5.9)	5 (1.4)	0	0
Paresthesia	18 (5.0)	6 (1.7)	0	0	10 (2.8)	2 (0.6)	0	0
Gamma-glutamyl transferase increased	16 (4.5)	5 (1.4)	1 (0.3)	0	4 (1.1)	5 (1.4)	0	0
Thrombocytopenia	15 (4.2)	6 (1.7)	2 (0.6)	0	12 (3.4)	3 (0.8)	1 (0.3)	0
Neutropenia	11 (3.1)	7 (1.9)	1 (0.3)	0	11 (3.1)	7 (2.0)	2 (0.6)	0
Pneumonia	4 (1.1)	4 (1.1)	0	0	3 (0.8)	1 (0.3)	0	0
Hyponatremia	2 (0.6)	4 (1.1)	0	0	0	2 (0.6)	0	0

^a^Any TRAE leading to discontinuation of study treatment due to the discontinuation of at least one medication (nivolumab, placebo, oxaliplatin, capecitabine, or tegafur–gimeracil–oteracil [S-1])

^b^Any TRAEs that led to delayed administration or a reduction in the dose of at least one medication (nivolumab, placebo, oxaliplatin, capecitabine, or tegafur–gimeracil–oteracil [S-1])

^c^TRAEs in ≥ 10% of patients in either group or Grade 3–5 TRAEs in ≥ 1% of patients in either group

*TRAE* treatment-related adverse event

## Discussion

Only a few studies have investigated the long-term (3 years) efficacy and safety of nivolumab in patients with advanced gastric cancer; ATTRACTION-2 assessed nivolumab as monotherapy (third-line or later) [20] and CheckMate 649 assessed nivolumab plus chemotherapy [18]. To our knowledge, this analysis of ATTRACTION-4, which was conducted in Japan, South Korea, and Taiwan, is the first report of a 3-year follow-up of nivolumab plus chemotherapy as first-line therapy in Asian patients with advanced gastric cancer.

The results reported here are consistent with those obtained at the prior data cutoff [11], further demonstrating the long-term effectiveness of combining nivolumab with oxaliplatin-based chemotherapy in terms of extending PFS and improving the ORR compared with placebo plus chemotherapy. Furthermore, we observed a tail plateau for PFS and DOR in the nivolumab plus chemotherapy group, a characteristic effect of immuno-oncology drugs. The benefit of nivolumab on PFS was apparent in most subgroups of patients, and was obtained regardless of whether SOX or CAPOX was chosen as chemotherapy.

Although OS did not differ between the two groups, we should acknowledge that the OS was relatively long in the placebo plus chemotherapy group, but a large proportion of patients received nivolumab as subsequent therapy in the placebo group, which might decrease the difference in short-term survival. This is because the investigators could request unblinding of the study treatment when starting a subsequent therapy because nivolumab is approved in Japan, Korea, and Taiwan for use as third-/later-line monotherapy in patients with advanced gastric/gastroesophageal junction cancer.

However, the landmark analysis at 1, 2, and 3 years showed that the OS HR improved over time. These findings may suggest that the long-term effectiveness differs between immuno-oncology drugs, such as nivolumab, and conventional chemotherapy. Furthermore, considering that a large proportion of patients in the chemotherapy group received nivolumab as the subsequent therapy and that 48.5% (32/66) of 3-year survivors received an immune checkpoint inhibitor as subsequent therapy in the placebo plus chemotherapy group, which was higher than in the nivolumab plus chemotherapy group (11.4% [9/79]), the favorable HR in the 3-year landmark analysis suggest that using nivolumab as first-line chemotherapy might provide a greater contribution to long-term survival more than its use as third-line chemotherapy. Biomarkers for predicting a long-term survival benefit should be established.

It is notable that the median DOR was approximately 5 months longer in the nivolumab plus chemotherapy group. Once CR was achieved, the 3-year survival rate of patients treated with nivolumab plus chemotherapy was almost 80%, compared with approximately 35% in patients treated with chemotherapy alone. In CheckMate 649, nivolumab plus chemotherapy provided a greater OS benefit vs. chemotherapy in responders than in non-responders, as revealed by an exploratory landmark analysis of patients treated with nivolumab plus chemotherapy [21]. In ATTRACTION-2, responders with PR or CR in the nivolumab group showed a favorable 3-year survival rate [20]. Therefore, greater long-term survival benefits can be obtained with immune checkpoint inhibitors compared with placebo, especially among patients with deep responses (i.e., CR).

The safety profile of nivolumab plus chemotherapy in this study was consistent with the results of the prior publication [11]. No new safety signals were identified. However, we should be aware that TRAEs occurred even after 2 years. Although Grade 3–4 and serious TRAEs were more frequent in the nivolumab plus chemotherapy group than in the placebo plus chemotherapy group, the TRAEs in the nivolumab plus chemotherapy group were manageable in accordance with the known safety management algorithm [22].

The results of ATTRACTION-4 in Asian patients should be discussed in the context of prior trials conducted in Asia and other regions. For example, in CheckMate 649, patients with advanced gastric, gastroesophageal junction, or esophageal adenocarcinoma were enrolled without restrictions on their cancer PD-L1 expression level, and were treated with nivolumab plus chemotherapy (CAPOX or FOLFOX), nivolumab plus ipilimumab, or chemotherapy alone [10, 17, 18]. In that trial, nivolumab plus chemotherapy improved OS compared with chemotherapy alone. This OS benefit was apparent in all randomized patients and patients with PD-L1 (CPS) of ≥ 1 or ≥ 5. In addition, longer term follow-ups showed that the clinically meaningful benefit of nivolumab plus chemotherapy on OS was maintained at 2 and 3 years [17, 18]. The 3-year follow-up of ATTRACTION-4, particularly the sustained effect of nivolumab plus chemotherapy on PFS and the signs of improved OS, coupled with an acceptable safety profile, support the results of CheckMate 649 and highlight the durable efficacy of this treatment.

Favorable efficacy of other immunotherapies combined with chemotherapy was also reported for pembrolizumab in KEYNOTE-859 [23]. Collectively, these findings indicate that combining an immunotherapy with chemotherapy confers prognostic advantages over chemotherapy alone in patients with advanced gastric/gastroesophageal junction cancer. Promising effects of other therapies, such as an anti-CLDN18.2 antibody and FGFR2 inhibitor, have also been reported [24–26]. Even if such treatments become available, the present long-term results of ATTRACTION-4 will serve as an important benchmark in the future.

## Limitations

The main limitations of the study, which were previously described, include the use of tumor cell PD-L1 expression rather than PD-L1 CPS, which may limit generalizability to settings where PD-L1 CPS is determined. This study only enrolled patients in Japan, South Korea, and Taiwan, which may limit its generalizability to other Asian countries. Nevertheless, the long-term efficacy and safety of nivolumab were comparable to those reported in CheckMate 649 [10, 17, 18]. We should also be cautious when interpreting the subgroup and landmark analyses because the trial was not specifically preplanned and powered for these analyses. There were some differences in PFS between investigator-assessed PFS and centrally assessed PFS. In this study, disease progression was assessed by the investigator and by a central committee, but the investigator was responsible for deciding whether or not treatment should be discontinued in the event of disease progression and whether a subsequent therapy should be started. Treatments could also be discontinued due to unacceptable toxicity or consent withdrawal, or continued following disease progression if agreed by the investigator and patient. Patients who received subsequent therapy after investigator-assessed disease progression but before centrally assessed disease progression were handled as censored patients in the central assessment of PFS. These factors may explain why the centrally assessed PFS was more favorable than the investigator-assessed PFS.

## Conclusion

In conclusion, the 3-year follow-up of ATTRACTION-4 demonstrated the long-term clinical benefit of nivolumab combined with chemotherapy in terms of prolonged PFS and DOR in patients with previously untreated gastric cancer. We have also demonstrated the long-term efficacy of nivolumab plus chemotherapy in terms of the improved HR of OS at 3 years and the high 3-year survival rate in patients achieving CR or PR. No new safety signals or major late-onset select TRAEs were observed for nivolumab plus chemotherapy.

## Supplementary Information

Below is the link to the electronic supplementary material.

Supplementary file1 (DOCX 86 KB)

## Data Availability

Qualified researchers may request Ono Pharmaceutical to disclose individual patient-level data from clinical studies through the following website: https://www.clinicalstudydatarequest.com/. For more information on Ono Pharmaceutical’s Policy for the Disclosure of Clinical Study Data, please see the following website: https://www.onopharma.com/en/company/policies/clinical_trial_data_transparency_policy.html

## References

[1] Ferlay J, Colombet M, Soerjomataram I, Parkin DM, Piñeros M, Znaor A, Bray F. Cancer statistics for the year 2020: an overview. Int J Cancer. 2021;149:778–89. 10.1002/ijc.33588. 10.1002/ijc.33588 33818764

[2] Wong MCS, Huang J, Chan PSF, Choi P, Lao XQ, Chan SM, et al. Global incidence and mortality of gastric cancer, 1980–2018. JAMA Netw Open. 2021;4(7):e2118457. 10.1001/jamanetworkopen.2021.18457. 34309666 10.1001/jamanetworkopen.2021.18457 PMC8314143

[3] Fuchs CS, Shitara K, Di Bartolomeo M, Lonardi S, Al-Batran SE, Van Cutsem E, et al. Ramucirumab with cisplatin and fluoropyrimidine as first-line therapy in patients with metastatic gastric or junctional adenocarcinoma (RAINFALL): a double-blind, randomised, placebo-controlled, phase 3 trial. Lancet Oncol. 2019;20(3):420–35. 10.1016/s1470-2045(18)30791-5. 30718072 10.1016/S1470-2045(18)30791-5

[4] Hecht JR, Bang YJ, Qin SK, Chung HC, Xu JM, Park JO, et al. Lapatinib in combination with capecitabine plus oxaliplatin in human epidermal growth factor receptor 2-positive advanced or metastatic gastric, esophageal, or gastroesophageal adenocarcinoma: TRIO-013/LOGiC–a randomized phase III trial. J Clin Oncol. 2016;34(5):443–51. 10.1200/jco.2015.62.6598. 26628478 10.1200/JCO.2015.62.6598

[5] Ryu MH, Baba E, Lee KH, Park YI, Boku N, Hyodo I, et al. Comparison of two different S-1 plus cisplatin dosing schedules as first-line chemotherapy for metastatic and/or recurrent gastric cancer: a multicenter, randomized phase III trial (SOS). Ann Oncol. 2015;26(10):2097–101. 10.1093/annonc/mdv316. 26216386 10.1093/annonc/mdv316

[6] Shah MA, Bang YJ, Lordick F, Alsina M, Chen M, Hack SP, et al. Effect of fluorouracil, leucovorin, and oxaliplatin with or without onartuzumab in HER2-negative, MET-positive gastroesophageal adenocarcinoma: the METGastric randomized clinical trial. JAMA Oncol. 2017;3(5):620–7. 10.1001/jamaoncol.2016.5580. 27918764 10.1001/jamaoncol.2016.5580 PMC5824210

[7] Yamada Y, Boku N, Mizusawa J, Iwasa S, Kadowaki S, Nakayama N, et al. Docetaxel plus cisplatin and S-1 versus cisplatin and S-1 in patients with advanced gastric cancer (JCOG1013): an open-label, phase 3, randomised controlled trial. Lancet Gastroenterol Hepatol. 2019;4(7):501–10. 10.1016/s2468-1253(19)30083-4. 31101534 10.1016/S2468-1253(19)30083-4

[8] Yamada Y, Higuchi K, Nishikawa K, Gotoh M, Fuse N, Sugimoto N, et al. Phase III study comparing oxaliplatin plus S-1 with cisplatin plus S-1 in chemotherapy-naïve patients with advanced gastric cancer. Ann Oncol. 2015;26(1):141–8. 10.1093/annonc/mdu472. 25316259 10.1093/annonc/mdu472

[9] Lee KW, Chung IJ, Ryu MH, Park YI, Nam BH, Oh HS, et al. Multicenter phase III trial of S-1 and cisplatin versus S-1 and oxaliplatin combination chemotherapy for first-line treatment of advanced gastric cancer (SOPP trial). Gastric Cancer. 2021;24(1):156–67. 10.1007/s10120-020-01101-4. 32596783 10.1007/s10120-020-01101-4

[10] Janjigian YY, Shitara K, Moehler M, Garrido M, Salman P, Shen L, et al. First-line nivolumab plus chemotherapy versus chemotherapy alone for advanced gastric, gastro-oesophageal junction, and oesophageal adenocarcinoma (CheckMate 649): a randomised, open-label, phase 3 trial. Lancet. 2021;398(10294):27–40. 10.1016/s0140-6736(21)00797-2. 34102137 10.1016/S0140-6736(21)00797-2 PMC8436782

[11] Kang YK, Chen LT, Ryu MH, Oh DY, Oh SC, Chung HC, et al. Nivolumab plus chemotherapy versus placebo plus chemotherapy in patients with HER2-negative, untreated, unresectable advanced or recurrent gastric or gastro-oesophageal junction cancer (ATTRACTION-4): a randomised, multicentre, double-blind, placebo-controlled, phase 3 trial. Lancet Oncol. 2022;23(2):234–47. 10.1016/s1470-2045(21)00692-6. 35030335 10.1016/S1470-2045(21)00692-6

[12] US Food & Drug Administration. FDA approves nivolumab in combination with chemotherapy for metastatic gastric cancer and esophageal adenocarcinoma. https://www.fda.gov/drugs/resources-information-approved-drugs/fda-approves-nivolumab-combination-chemotherapy-metastatic-gastric-cancer-and-esophageal. Accessed 21 Feb 2024.

[13] Bristol Myers Squibb. Bristol Myers Squibb receives European Commission approval for Opdivo (nivolumab) + chemotherapy for patients with HER2 negative, advanced or metastatic gastric, gastroesophageal junction or esophageal adenocarcinoma. https://news.bms.com/news/corporate-financial/2021/Bristol-Myers-Squibb-Receives-European-Commission-Approval-for-Opdivo-nivolumab--Chemotherapy-for-Patients-with-HER2-Negative-Advanced-or-Metastatic-Gastric-Gastroesophageal-Junction-or-Esophageal-Adenocarcinoma-/default.aspx. Accessed 21 Feb 2024.

[14] National Comprehensive Cancer Network. NCCN Clinical Practice Guidelines in Oncology (NCCN Guidelines®). Gastric Cancer. Version 1.2023 — March 10, 2023. https://www.nccn.org/professionals/physician_gls/pdf/gastric.pdf. Accessed 10 July 2023.

[15] Japanese Gastric Cancer Association. Japanese Gastric Cancer Treatment Guidelines 2021 (6th edition). Gastric Cancer. 2023;26(1):1–25. 10.1007/s10120-022-01331-8. 36342574 10.1007/s10120-022-01331-8 PMC9813208

[16] Kim TH, Kim IH, Kang SJ, Choi M, Kim BH, Eom BW, et al. Korean Practice Guidelines for Gastric Cancer 2022: an evidence-based, multidisciplinary approach. J Gastric Cancer. 2023;23(1):3–106. Erratum J Gastric Cancer. 2023;23(2):365–373. 10.5230/jgc.2023.23.e11. 10.5230/jgc.2023.23.e11 PMC9911619 36750993

[17] Shitara K, Ajani JA, Moehler M, Garrido M, Gallardo C, Shen L, et al. Nivolumab plus chemotherapy or ipilimumab in gastro-oesophageal cancer. Nature. 2022;603(7903):942–8. 10.1038/s41586-022-04508-4. 35322232 10.1038/s41586-022-04508-4 PMC8967713

[18] Janjigian YY, Shitara K, Moehler MH, Garrido M, Gallardo C, Shen L, et al. Nivolumab (NIVO) plus chemotherapy (chemo) vs chemo as first-line (1L) treatment for advanced gastric cancer/gastroesophageal junction cancer/esophageal adenocarcinoma (GC/GEJC/EAC): 3-year follow-up from CheckMate 649. J Clin Oncol. 2023;41(4_suppl):291. 10.1200/JCO.2023.41.4_suppl.291.37713657

[19] Eyck BM, van Lanschot JJB, Hulshof M, van der Wilk BJ, Shapiro J, van Hagen P, et al. Ten-year outcome of neoadjuvant chemoradiotherapy plus surgery for esophageal cancer: the randomized controlled CROSS trial. J Clin Oncol. 2021;39(18):1995–2004. 10.1200/jco.20.03614. 33891478 10.1200/JCO.20.03614

[20] Boku N, Satoh T, Ryu MH, Chao Y, Kato K, Chung HC, et al. Nivolumab in previously treated advanced gastric cancer (ATTRACTION-2): 3-year update and outcome of treatment beyond progression with nivolumab. Gastric Cancer. 2021;24(4):946–58. 10.1007/s10120-021-01173-w. 33743112 10.1007/s10120-021-01173-w PMC8205916

[21] Yamaguchi K, Janjigian YY, Jaffer AA, Moehler M, Garrido M, Gallardo C, et al. First-line nivolumab plus chemotherapy vs chemotherapy for advanced gastric cancer/gastroesophageal junction cancer/esophageal adenocarcinoma: survival analyses by tumor response from CheckMate 649. In: Oral presentation, SSY3–4–2. 60th Annual Meeting of Japan Society of Clinical Oncology, Kobe, Japan, October 20–22, 2022.

[22] Ono Pharma, Bristol Myers Squibb. Opdivo® (nivolumab) 20mg, 100mg, 120mg, 240mg, intravenous injection. Proper Use Guide (Single Agent), July 2023. Available at: https://www.opdivo.jp/download/materials/opdivo/14456/OPD-T10045E_202307.pdf. Accessed 18 Sep 2023 (In Japanese).

[23] Rha SY, Wyrwicz LS, Weber PEY, Bai Y, Ryu MH, Lee J, et al. VP1-2023: pembrolizumab (pembro) plus chemotherapy (chemo) as first-line therapy for advanced HER2-negative gastric or gastroesophageal junction (G/GEJ) cancer: phase III KEYNOTE-859 study. Ann Oncol. 2023;34(3):319–20. 10.1016/j.annonc.2023.01.006.

[24] Shitara K, Lordick F, Bang YJ, Enzinger P, Ilson D, Shah MA, et al. Zolbetuximab plus mFOLFOX6 in patients with CLDN18.2-positive, HER2-negative, untreated, locally advanced unresectable or metastatic gastric or gastro-oesophageal junction adenocarcinoma (SPOTLIGHT): a multicentre, randomised, double-blind, phase 3 trial. Lancet. 2023;401(10389):1655–68. 10.1016/s0140-6736(23)00620-7. 37068504 10.1016/S0140-6736(23)00620-7

[25] Xu R-H, Shitara K, Ajani JA, Bang YJ, Enzinger PC, Ilson DH, et al. Zolbetuximab + CAPOX in 1L claudin-182+ (CLDN182+)/HER2− locally advanced (LA) or metastatic gastric or gastroesophageal junction (mG/GEJ) adenocarcinoma: primary phase 3 results from GLOW. J Clin Oncol. 2023. 10.1200/JCO.2023.41.36_suppl.405736.38127780

[26] Wainberg ZA, Enzinger PC, Kang YK, Qin S, Yamaguchi K, Kim IH, et al. Bemarituzumab in patients with FGFR2b-selected gastric or gastro-oesophageal junction adenocarcinoma (FIGHT): a randomised, double-blind, placebo-controlled, phase 2 study. Lancet Oncol. 2022;23(11):1430–40. 10.1016/s1470-2045(22)00603-9. 36244398 10.1016/S1470-2045(22)00603-9

