# Supplementary material for: Nivolumab plus chemotherapy in patients with HER2-negative, previously untreated, unresectable, advanced, or recurrent gastric/gastroesophageal junction cancer: 3-year follow-up of the ATTRACTION-4 randomized, double-blind, placebo-controlled, phase 3 trial
Source: Gastric Cancer. 2024 Aug 20;27(6):1287–301. doi: 10.1007/s10120-024-01535-0 (PMC11513732; doi:10.1007/s10120-024-01535-0)

# Electronic Supplementary materials: Online resource 1

**Nivolumab plus chemotherapy in patients with HER2-negative, previously untreated, unresectable, advanced, or recurrent gastric/gastroesophageal junction cancer: 3-year follow-up of the ATTRACTION-4 randomized, double-blind, placebo-controlled, phase 3 trial**

**Corresponding author**

Dr. Yoon-Koo Kang

Email: [ykkang@amc.seoul.kr](mailto:ykkang@amc.seoul.kr)

| **Contents** | **Page** |
| --- | --- |
| **Supplementary Table 1** Duration of treatment and reasons for discontinuation of study treatment | 2–3 |
| **Supplementary Fig. 1** Patient disposition | 4 |
| **Supplementary Fig. 2** Timing of treatment-related adverse events with a potential immunologic etiology in the nivolumab plus chemotherapy (a) and placebo plus chemotherapy (b) groups | 5 |

**Supplementary Table 1** Duration of treatment and reasons for discontinuation of study treatment

|  | Nivolumab plus chemotherapy  *N* = 359 | | | Placebo plus chemotherapy  *N* = 358 | | |
| --- | --- | --- | --- | --- | --- | --- |
|  | All patients | Nivolumab plus SOX  *N* = 229 | Nivolumab plus CAPOX  *N* = 130 | All patients | Placebo plus SOX  *N* = 230 | Placebo plus CAPOX  *N* = 128 |
| Duration of treatment, months, median (range) |  |  |  |  |  |  |
| Nivolumab or placebo | 5.9 (0.0–47.7) | 6.3 (0.0–47.1) | 5.2 (0.0–47.7) | 5.2 (0.0–48.0) | 5.0 (0.0–45.3) | 5.7 (0.0–48.0) |
| Oxaliplatin | 5.3 (0.0–36.1) | 5.4 (0.0–36.1) | 5.0 (0.0–32.2) | 4.6 (0.0–37.3) | 4.6 (0.0–37.3) | 4.9 (0.0–29.2) |
| S-1 | 7.1 (0.1–47.6) | 7.1 (0.1–47.6) | – | 5.4 (0.1–45.8) | 5.4 (0.1–45.8) | – |
| Capecitabine | 6.0 (0.1–48.0) | – | 6.0 (0.1–48.0) | 6.1 (0.2–48.5) | – | 6.1 (0.2–48.5) |
|  | All patients | | | All patients | | |
| Discontinuation of nivolumab or placebo, *n* (%) | 341 (95.0) | | | 349 (97.5) | | |
| Reason for discontinuation of nivolumab or placebo, *n* (%) (one reason per patient) | | | | | | |
| PD according to RECIST guidelines (version 1.1) | 219 (61.0) | | | 260 (72.6) | | |
| Worsening of clinical symptoms due to disease progression | 20 (5.6) | | | 19 (5.3) | | |
| Unacceptable toxicity | 31 (8.6) | | | 16 (4.5) | | |
| Investigator decision | 28 (7.8) | | | 11 (3.1) | | |
| Other | 43 (12.0) | | | 43 (12.0) | | |
| Subsequent therapies, *n* (%) (multiple treatments possible) |  | | |  | | |
| Radiotherapy | 30 (8.4) | | | 30 (8.4) | | |
| Surgery | 35 (9.7) | | | 28 (7.8) | | |
| Drug therapies ^a^ | 241 (67.1) | | | 249 (69.6) | | |
| Paclitaxel | 209 (58.2) | | | 223 (62.3) | | |
| Ramucirumab | 165 (46.0) | | | 189 (52.8) | | |
| Irinotecan | 80 (22.3) | | | 88 (24.6) | | |
| Immune checkpoint inhibitors | 47 (13.1) | | | 117 (32.7) | | |
| Nivolumab | 40 (11.1) | | | 110 (30.7) | | |
| Pembrolizumab | 7 (1.9) | | | 9 (2.5) | | |
| Drainage | 45 (12.5) | | | 52 (14.5) | | |
| Others | 77 (21.4) | | | 79 (22.1) | | |

^a^ Only three most common pharmacotherapies are listed

*CAPOX* capecitabine plus oxaliplatin, *PD* progressive disease, *RECIST* Response Evaluation Criteria in Solid Tumors, *S-1* tegafur–gimeracil–oteracil potassium, *SOX* S-1 (tegafur–gimeracil–oteracil potassium) plus oxaliplatin

**Supplementary Fig. 1** Patient disposition


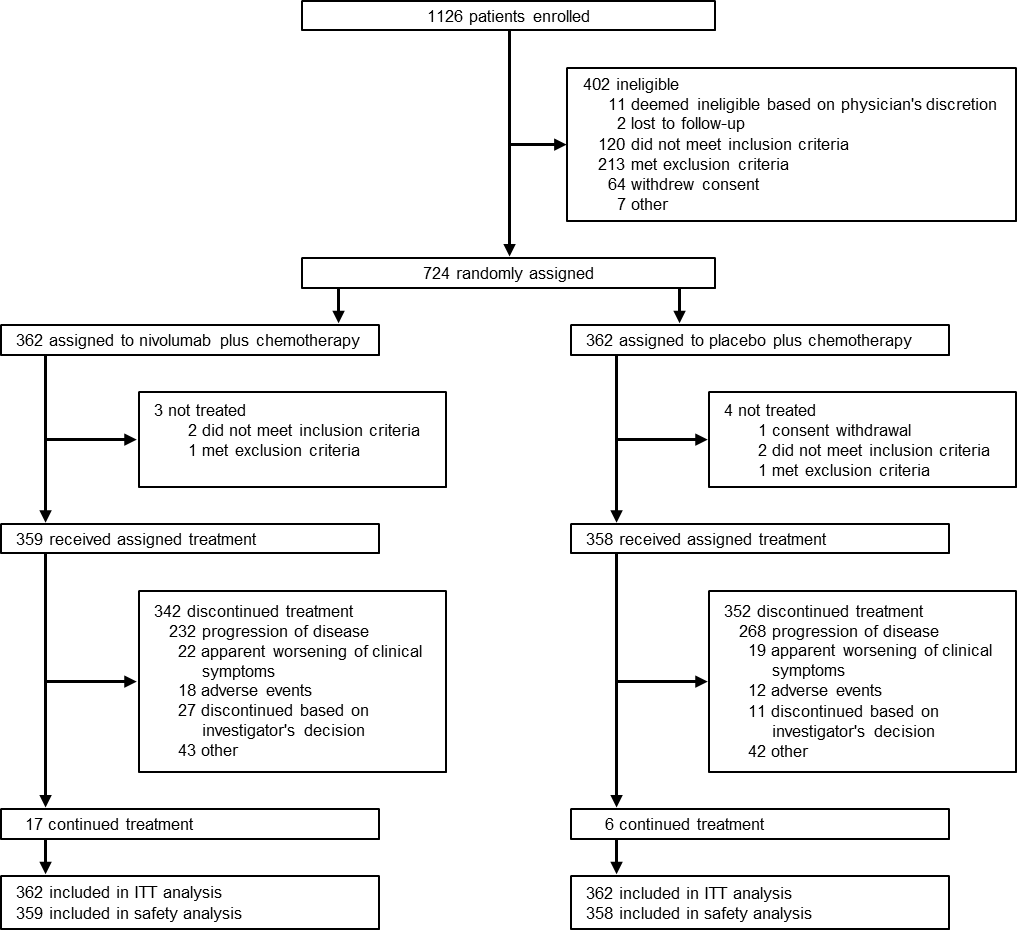


*ITT* intention-to-treat

**Supplementary Fig. 2** Timing of treatment-related adverse events with a potential immunologic etiology in the nivolumab plus chemotherapy (a) and placebo plus chemotherapy (b) groups


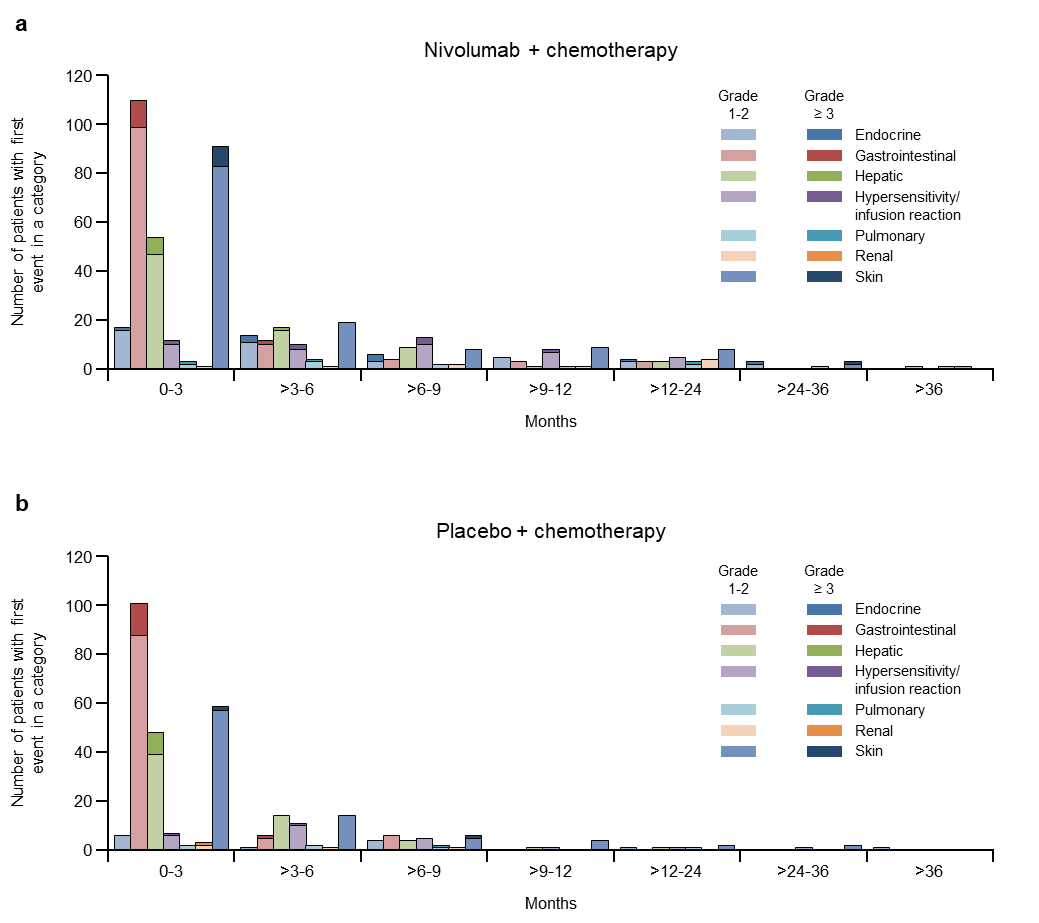

Supplement: Supplementary file 1 — Supplementary file1 (DOCX 86 KB) [file 10120_2024_1535_MOESM1_ESM.docx]
